# Association of residual feed intake with abundance of ruminal bacteria and biopolymer hydrolyzing enzyme activities during the peripartal period and early lactation in Holstein dairy cows

**DOI:** 10.1186/s40104-018-0258-9

**Published:** 2018-05-14

**Authors:** Ahmed A. Elolimy, José M. Arroyo, Fernanda Batistel, Michael A. Iakiviak, Juan J. Loor

**Affiliations:** 1 Mammalian NutriPhysioGenomics, Department of Animal Sciences, University of Illinois, Urbana, IL USA; 2 Department of Animal Sciences, University of Illinois, Urbana, IL USA; 3 Departamento de Nutrición Animal, Instituto de Producción Animal, Facultad de Veterinaria, Universidad de la Republica, Ruta 1 km 42.5, 80100 San José, Uruguay; 4 Division of Nutritional Sciences, Illinois Informatics Institute, University of Illinois, Urbana, IL USA

**Keywords:** Dairy cows, Enzyme activity, Peripartal period, RFI, Rumen bacteria

## Abstract

**Background:**

Residual feed intake (RFI) in dairy cattle typically calculated at peak lactation is a measure of feed efficiency independent of milk production level. The objective of this study was to evaluate differences in ruminal bacteria, biopolymer hydrolyzing enzyme activities, and overall performance between the most- and the least-efficient dairy cows during the peripartal period. Twenty multiparous Holstein dairy cows with daily ad libitum access to a total mixed ration from d − 10 to d 60 relative to the calving date were used. Cows were classified into most-efficient (i.e. with low RFI, *n* = 10) and least-efficient (i.e. with high RFI, *n* = 10) based on a linear regression model involving dry matter intake (DMI), fat-corrected milk (FCM), changes in body weight (BW), and metabolic BW.

**Results:**

The most-efficient cows had ~ 2.6 kg/d lower DMI at wk 4, 6, 7, and 8 compared with the least-efficient cows. In addition, the most-efficient cows had greater relative abundance of total ruminal bacterial community during the peripartal period. Compared with the least-efficient cows, the most-efficient cows had 4-fold greater relative abundance of *Succinivibrio dextrinosolvens* at d − 10 and d 10 around parturition and tended to have greater abundance of *Fibrobacter succinogenes* and *Megaspheara elsdenii*. In contrast, the relative abundance of *Butyrivibrio proteoclasticus* and *Streptococcus bovis* was lower and *Succinimonas amylolytica* and *Prevotella bryantii* tended to be lower in the most-efficient cows around calving. During the peripartal period, the most-efficient cows had lower enzymatic activities of cellulase, amylase, and protease compared with the least-efficient cows.

**Conclusions:**

The results suggest that shifts in ruminal bacteria and digestive enzyme activities during the peripartal period could, at least in part, be part of the mechanism associated with better feed efficiency in dairy cows.

**Electronic supplementary material:**

The online version of this article (10.1186/s40104-018-0258-9) contains supplementary material, which is available to authorized users.

## Background

Improving feed efficiency in dairy cows has become increasingly important for the dairy industry since feed expenses are the most costly component of dairy systems. Therefore, identifying and selecting for dairy cows that use feed efficiently, i.e. require less feed for maintenance and for the same level of milk production, provides opportunities for reducing production costs and maximizing the economic returns for dairy producers. Residual feed intake (RFI) has been used in dairy cows to define feed efficiency independent of body size and milk production level [1, 2]. Residual feed intake is calculated as the difference between the actual and the predicted feed consumption of individual dairy cows after adjusting the DMI for the level of production through a linear regression model [3]. Hence, the most-efficient dairy cows, i.e. with a negative RFI, consume less feed than expected for their production level compared with the least-efficient dairy cows having a positive RFI [4].

Phenotypic variation in RFI between individual animals is directly related to variation in feed digestion, efficiency of nutrient use, and microbial protein production, all of which take place in the rumen, suggesting a vital role for the rumen in improving feed efficiency [5]. The rumen harbors a complex anaerobic microbial community, mainly bacteria, capable of producing various biopolymer hydrolyzing enzymes (e.g., amylase, xylanase, cellulase, and protease) that convert low-quality feed consumed into energy- and protein-rich compounds for the host [6]. Production of VFA and microbial protein can supply the ruminant with 70% and 50% of its daily energy and protein requirements, respectively [7].

To the best of our knowledge, the only available study on the association between rumen bacteria and RFI phenotypes in lactating dairy cows was conducted by Jewell et al. [8] who observed that ruminal bacteria profiles in the most-efficient lactating dairy cows differed from the least-efficient ones; for example, over the course of two lactations, the higher abundance of bacterial genera *Anaerovibrio* and *Butyrivibrio* were associated with the least-efficient cows. That study only focused on profiling the composition of rumen bacterial community between the most- and the least-efficient lactating dairy cows and did not provide information on the microbial enzyme activities in the rumen between the two RFI phenotypes. Therefore, it is reasonable to hypothesize that shifts in bacterial composition could be accompanied by changes in major microbial enzyme activities and impact RFI phenotypes.

The “peripartal” period in dairy cows is characterized by a marked negative energy and metabolizable protein balance at least in part due to the decrease in voluntary DMI and the high requirements for nutrients by the fetus and lactating mammary gland. Therefore, the peripartal period is challenging for dairy cows resulting in higher susceptibility for developing metabolic disorders [9–11]. Current nutritional management of peripartal cows encompasses the feeding of higher-concentrate diets postcalving to provide the rumen bacterial communities with a more readily-available source of energy. As a result, bacterial composition in the rumen changes relative to the dry period [12–14]. Although various studies were recently conducted to evaluate shifts in rumen bacterial communities during the peripartal period in dairy cows, little attention has been given to changes in microbial enzyme activities. Clearly, it is possible that changes in the bacterial community composition of the rumen during the peripartal period may contribute to differences in the major biopolymer hydrolyzing enzyme activities.

The current study aimed to evaluate abundance of selected ruminal bacterial species and activities of enzymes associated with protein and carbohydrate metabolism between cows classified as most-efficient (i.e., with negative RFI) and least-efficient (i.e., with positive RFI) using data collected from d − 10 to d 60 relative to the calving date. An important goal was to determine potential linkages between RFI phenotype, ruminal microorganisms, digestive enzyme activities, and overall performance.

## Methods

All experimental procedures were approved by The Institutional Animal Care and Use Committee at the University of Illinois (protocol number 14270).

### Animals, experimental design, and diets

A subset of 20 multiparous Holstein cows from a larger cohort were used [15]. Before calving, cows were housed in a freestall barn equipped with electronic recognition feeding system for each cow (American Calan Inc., Northwood, NH), while cows were housed in a tie-stall system during lactation. Cows were fed individually a TMR and allowed free access to feed and water at all times. The ration was formulated to meet cow predicted requirements according to NRC [16]. The feed ingredients for the close-up (from d − 28 to calving), fresh (from 1 to 30 DIM), and high-producing (from 31 to 60 DIM) TMR diets are shown in Table 1. Feed offered and refused were measured daily to calculate feed intake throughout the entire study. Weekly samples of the diets were collected to determine the DM content. Samples of the TMR and feed ingredients were stored frozen at − 20 °C and composited monthly for analyses of crude protein [[17]; method 990.03], NDF with heat-stable α-amylase and sodium sulfite [18], ADF [18], and ether extract [[17]; method 2003.05] by Cumberland Valley Analytical Services (Hagerstown, MD). The nutrient analysis of the diets is shown in Table 1. Body weight was recorded weekly during the entire feeding period.

**Table 1:** Ingredient composition and nutrient analysis of close-up (from d − 28 to parturition), fresh (from 1 to 30 DIM), and high-producing (from 31 to 60 DIM) diets

Item	Close-up	Fresh	High-producing
Ingredient composition, % of DM			
Alfalfa haylage	6.55	7.81	10.8
Corn silage	26.6	31.0	31.9
Wheat straw	26.5	3.25	–
Corn grain, ground, dry	12.6	22.21	20.7
Cottonseed	–	2.17	1.83
Molasses, beet sugar	4.03	5.50	4.51
Soybean hulls	3.46	4.25	9.96
Soybean meal, 48% CP	7.83	10.1	7.98
Expeller soybean meal^a^	5.80	5.16	5.17
Protein supplement^b^	0.78	1.81	1.58
Urea	0.59	0.39	0.40
Soychlor^c^	1.23	–	–
Saturated fat supplement^d^	–	2.25	2.14
Limestone	–	1.41	0.96
Salt	–	0.02	0.04
Dicalcium phosphate	0.52	1.17	0.92
Magnesium oxide	–	0.08	0.04
Magnesium sulfate	2.08	0.02	–
Sodium bicarbonate	–	0.84	0.59
Mineral vitamin mix^e^	0.17	0.17	0.20
Vitamin A^f^	0.03	0.02	0.02
Vitamin D^g^	0.03	–	–
Vitamin E^h^	0.60	–	–
Biotin^i^	0.70	0.42	0.32
Nutrient analysis, % of DM			
CP	15.6 ± 0.32	17.7 ± 0.36	17.4 ± 0.36
NDF	40.8 ± 0.68	29.2 ± 0.59	31.4 ± 0.62
ADF	27.5 ± 0.50	19.5 ± 0.38	21.5 ± 0.48
NFC	34.9 ± 0.81	41.4 ± 0.55	40.7 ± 0.54
Ether extract	2.32 ± 0.05	5.12 ± 0.14	5.13 ± 0.14

^a^SoyPlus, West Central Soy (Ralston, IA)

^b^ProVAAl AADvantage, Perdue AgriBusiness (Salisbury, MD)

^c^West Central Soy

^d^Energy Booster 100, Milk Specialties Global (Eden Prairie, MN)

^e^Contained a minimum of 5% Mg, 10% S, 7.5% K, 2.0% Fe, 3.0% Zn, 3.0% Mn, 5,000 mg/kg Cu, 250 mg/kg I, 40 mg/kg Co, 150 mg/kg Se, 2,200 kIU/kg vitamin A, 660 kIU/kg vitamin D_3_, and 7,700 IU/kg vitamin E

^f^Contained 30,000 kIU/kg

^g^Contained 5,000 kIU/kg

^h^Contained 44,000 kIU/kg

^i^ADM Animal Nutrition (Quincy, IL)

### Sample collection

Cows were automatically milked 3 times daily, and individual milk production recorded daily. Consecutive morning, midday, and evening milk samples were collected once a week, and stored at 4 °C for fat analysis by an infrared system (Dairy Lab Services, Dubuque, IA). The fat-corrected milk (FCM) yield for each cow was calculated according to NRC (2001) equations.

Although cannulation has been previously considered as the standard method collection of a representative sample of ruminal contents [19], performing surgical cannulations and its associated post-surgical costs limit its application in large groups of cows and often leads to reduced statistical power [20, 21]. The lack of significant differences in ruminal fermentation and microbiota composition between samples harvested via cannula or stomach tubing demonstrated that the latter is suitable for ruminal digesta sampling [20, 22–24]. Therefore, stomach tubing was deemed suitable and allowed us to use a greater number of cows. Four-hundred mL of mixed ruminal contents was collected from each cow 4 h postfeeding via stomach tubing at d − 10 before expected calving date and at 10, 30, and 60 DIM. Ruminal contents were immediately frozen in liquid nitrogen, transported to the laboratory and stored at − 80 °C for later analysis.

### Residual feed intake calculation

**Fig. 1:**
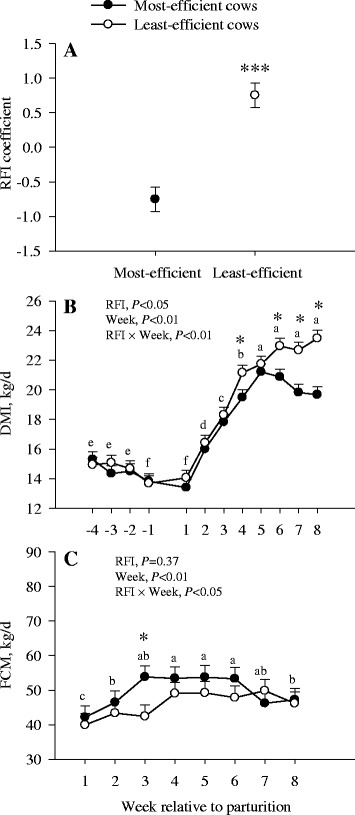
RFI coefficients (**a**), DMI (**b**), and fat-corrected milk (FCM) (**c**) in the most- and the least-efficient multiparous Holstein dairy cows during the peripartal period. Significant differences between RFI groups are denoted with an asterisk (*P < 0.05 or *** *P* < 0.0001). ^a-e^Different letters indicate differences due to the main effect of time (*P* < 0.05)

The RFI (kg/d) for the 20 cows used from the bigger cohort in the experiment of Batistel et al. [15] was calculated using individual data from the entire period between − 28 d through 60 d around parturition. This number of cows gave us a complete set of samples across the chosen time points, i.e. for ruminal fluid and production. Thus, the calculated RFI for the 20 cows allowed us to divide them into two groups based on their divergent in feed efficiency: the most-efficient cows with desirable (i.e. more negative) RFI coefficient vs. the least-efficient cows with unfavorable (i.e. more positive) RFI coefficient. The RFI coefficients were computed as the difference between the actual and the predicted DMI, where the predicted DMI was computed through a linear regression model using the regression of actual DMI on FCM, metabolic BW, and ADG as described previously [3]. The RFI coefficients for the most-efficient (*n* = 10) and the least-efficient (*n* = 10) cows are depicted in Fig. 1a.

### Ruminal bacteria DNA extraction and RT-PCR amplification

The total genomic DNA was isolated using the repeated bead-beating plus column (RBB + C) purification method described by Yu and Morrison [25] for mechanical lysis of bacterial cell wall employing the QIAamp DNA mini kit (QIAGEN) for DNA purification. This method has been applied in several hundred studies to extract a high yield microbial DNA from rumen contents [21]. The DNA quantity and quality were checked using 0.8% (*w*/*v*) agarose gel electrophoresis and NanoDrop spectrophotometer (ND 1000, NanoDrop Technologies, Inc., Wilmington, DE, USA) at 260 nm. Extracted DNA was standardized to 8 ng/μL for RT-PCR.

Primers were selected to amplify 10 major ruminal bacteria species that play key roles in cellulose and hemicellulose digestion, xylan degradation, proteolysis, propionate production, lactate utilization and ruminal biohydrogenation. The chosen primers along with 3 universal primers are listed in Table 2. A total of 10 μL of RT-PCR mixture contained 4 μL sample DNA, 5 μL 1× SYBR Green with ROX (Quanta BioSciences, Gaithersburg, MD, USA), 0.4 μL each of 10 μmol/L forward and reverse primers, and 0.2 μL DNase/RNase free water in a MicroAmpTM Optical 384-Well Reaction Plate (Applied Biosystems, Foster City, CA, USA). Negative controls without template DNA, standards, and samples were run on the same plate in triplicate. The RT-PCR reactions were performed with QuantStudio-7 Real-Time PCR instrument (ThermoFisher Scientific, USA) using the following program: initial denaturation at 95 °C for 5 min, followed by 40 cycles of 1 s at 95 °C and 30 s annealing at 60 °C. A dissociation stage was performed to determine the specificity of the amplification. Relative abundance of bacterial species was calculated using the geometric mean of the universal primers eubacterial primer 1 and eubacterial primer 2 (Table 2) [26, 27] with the efficiency-corrected Δ^−CT^ method [28]. The copy number of total bacterial 16S rRNA genes was measured to estimate the total bacterial population using RT-PCR analysis with eubacterial primer 3 (Table 2) [29] following the procedures described previously by Zhou et al. [30].

**Table 2:** Species-specific primers for the quantification of selected rumen bacterial populations using a real-time qPCR assay

Target bacterial species		Primer sequence (5` → 3`)	Reference	Efficiency^a^, %
*Anaerovibrio lipolytica*	F:^b^ R:^c^	GAAATGGATTCTAGTGGCAAACG ACATCGGTCATGCGACCAA	[13]	96.06
*Butyrivibrio proteoclasticus*	F: R:	GGGCTTGCTTTGGAAACTGTT CCCACCGATGTTCCTCCTAA	[13]	100.00
*Eubacterium ruminantium*	F: R:	CTCCCGAGACTGAGGAAGCTTG GTCCATCTCACACCACCGGA	[53]	106.08
*Fibrobacter succinogenes*	F: R:	GCGGGTAGCAAACAGGATTAGA CCCCCGGACACCCAGTAT	[53]	100.67
*Megaspheara elsdenii*	F: R:	AGATGGGGACAACAGCTGGA CGAAAGCTCCGAAGAGCCT	[53]	101.35
*Prevotella bryantii*	F: R:	AGCGCAGGCCGTTTGG GCTTCCTGTGCACTCAAGTCTGAC	[53]	105.03
*Selenomonas ruminantium*	F: R:	CAATAAGCATTCCGCCTGGG TTCACTCAATGTCAAGCCCTGG	[53]	97.91
*Succinimonas amylolytica*	F: R:	CGTTGGGCGGTCATTTGAAAC CCTGAGCGTCAGTTACTATCCAGA	[54]	96.80
*Streptococcus bovis*	F: R:	TTCCTAGAGATAGGAAGTTTCTTCGG ATGATGGCAACTAACAATAGGGGT	[53]	103.89
*Succinivibrio dextrinosolvens*	F: R:	TAGGAGCTTGTGCGATAGTATGG CTCACTATGTCAAGGTCAGGTAAGG	[54]	96.80
Eubacterial primer 1	F: R:	GGATTAGATACCCTGGTAGT CACGACACGAGCTGACG	[27]	95.26
Eubacterial primer 2	F: R:	GTGSTGCAYGGYTGTCGTCA ACGTCRTCCMCACCTTCCTC	[26]	95.30
Eubacterial primer 3	F:	CCTACGGGAGGCAGCAG	[29]	99.30
	R:	ATTACCGCGGCTGCTGG		

^a^Measured efficiencies of the primers in the qPCR reactions

^b^Forward primer

^c^Reverse primer

### Enzyme activities

Details of ruminal enzymatic assays for determining amylase, xylanase, cellulase, and protease enzymatic activities are reported in detail in the Additional file 1.

### Statistical analysis

The MIXED procedure of SAS (SAS Institute Inc., Cary, NC) was used for repeated measures analysis of DMI, FCM, bacterial abundance, and enzyme activities. The fixed effects in the model were RFI and time (week or day), and the random effect was cow. Significance was determined at *P* ≤ 0.05, and tendencies were determined at *P* ≤ 0.10.

## Results

### Production

The most-efficient cows had lower (*P* = 0.04) DMI at wk 4, 6, 7, and 8 (average = 2.6 kg/d) (Fig. 1b). Dry matter intake in both groups decreased (*P* < 0.01) from − 4 wk to calving but progressively increased (*P* < 0.01) postpartum. No differences (*P* = 0.37) between RFI groups were observed for FCM (Fig. 1c). A RFI × week (*P* = 0.02) was observed for FCM yield due to a greater increase (*P* < 0.01) in the most-efficient cows at wk 3. Both groups of cows increased FCM yield (*P* < 0.01) after calving.

### Relative abundance of bacteria

**Fig. 2:**
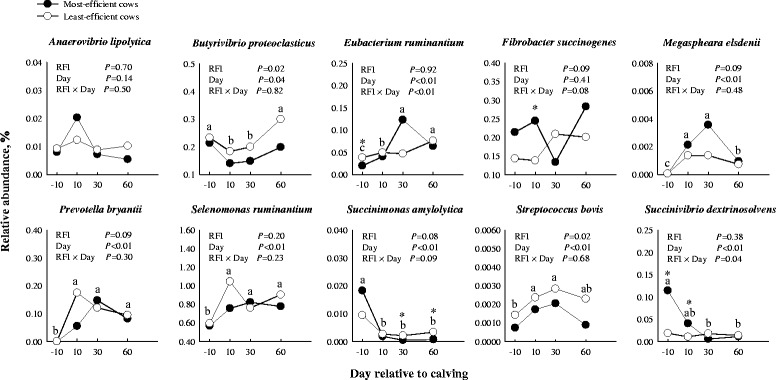
Relative abundance of 10 rumen bacterial species in the most- and the least-efficient multiparous Holstein dairy cows during the peripartal period. Significant differences (*P* < 0.05) between RFI groups at a given time point are denoted with an asterisk (*). ^a-c^Different letters indicate differences due to the main effect of time (*P* < 0.05). Data were logit transformed to ensure normality of residuals

The relative abundance of target ruminal bacteria species between the most- and the least-efficient cows during the peripartal period are depicted in Fig. 2. Results indicate that *Selenomonas ruminantium* was the most-abundant bacteria among the 10 analyzed, averaging 0.8% of 16S rRNA copy number. There were no RFI, day or RFI × day effects observed for abundance of *Anaerovibrio lipolytica*. A RFI × week interaction (*P* = 0.04) was observed for *Succinivibrio dextrinosolvens* due to a greater relative abundance in the most-efficient compared with least-efficient cows of ~ 6-fold at d − 10 and ~ 4-fold at d 10. Similarly, the most-efficient cows tended (*P* = 0.09) to have a greater overall abundance of *Fibrobacter succinogenes* and *Megaspheara elsdenii*, whereas a tendency (*P* = 0.08) for RFI × day interaction in the abundance of *Fibrobacter succinogenes* was due to greater levels of *Fibrobacter succinogenes* in the most-efficient cows at all days except d 30. There was no RFI × day (*P* = 0.48) effect for *Megaspheara elsdenii*.

Compared with the least-efficient cows, the abundance of *Butyrivibrio proteoclasticus* and *Streptococcus bovis* was lower (*P* = 0.02) in the most-efficient cows during the peripartal period. In addition, the most-efficient cows had lower (*P* < 0.01) overall abundance of *Eubacterium ruminantium* only at − 10 d. In addition, the most-efficient cows tended to have a lower abundance of *Succinimonas amylolytica* (*P* = 0.08) and *Prevotella bryantii* (*P* = 0.09) during the peripartal period. A tendency for a RFI × day (*P* = 0.09) effect in *Succinimonas amylolytica* was due to the most-efficient cows having a greater relative abundance at d − 10 followed by a decrease at d 30 and d 60 postpartum in both RFI groups. No RFI × day interaction (*P* = 0.30) was observed for *Prevotella bryantii*.

Several shifts in the bacterial populations were observed over time. For example, the relative abundance of *Eubacterium ruminantium*, *Prevotella bryantii*, *Selenomonas ruminantium*, and *Streptococcus bovis* was higher (*P* < 0.01) in both RFI groups after parturition. In contrast, *Megaspheara elsdenii* abundance decreased (*P* < 0.01) at d 60 compared with d 10 and d 30. The relative abundance of *Succinimonas amylolytica* was lower (*P* < 0.01) postpartum in both RFI groups while *Succinivibrio dextrinosolvens* was lower (*P* < 0.01) in the most-efficient group postpartum. *Butyrivibrio proteoclasticus* had low relative abundance (*P* = 0.04) at d 10 and d 30 postpartum, and abundance returned to prepartum values at d 60.

**Fig. 3:**
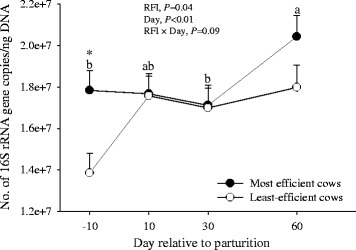
16S rRNA gene copy number of the total rumen bacterial community in rumen contents in the most- and the least-efficient multiparous Holstein dairy cows during the peripartal period. Significant differences (*P* < 0.05) between RFI groups at a given time point are denoted with an asterisk (*). ^a, b^Different letters indicate differences due to the main effect of time (*P* < 0.05)

The 16S rRNA copy numbers of the total ruminal bacterial community detected in the present study are shown in Fig. 3. The results indicate that the most-efficient cows had a greater (*P* = 0.04) relative abundance of bacteria compared with the least-efficient cows. However, there was a tendency for an RFI × day (*P* = 0.09) effect due to a greater bacterial community copy number in the rumen of the most-efficient cows at − 10 and 60 d, and a lower bacterial community copy number in the least-efficient cows at d 10.

### Digestive enzyme activities

**Fig. 4:**
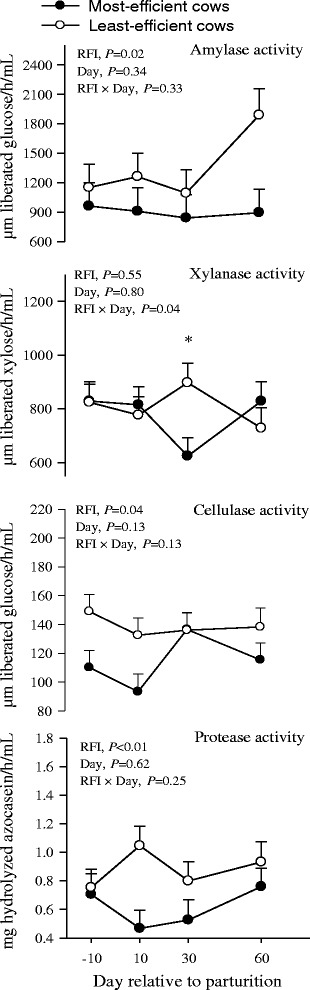
Activities of amylase, xylanase, cellulase, and protease in rumen contents from the most- and the least-efficient multiparous Holstein dairy cows during the peripartal period. Significant differences (*P* < 0.05) between RFI groups at a given time point are denoted with an asterisk (*)

The microbial enzyme activities in the rumen of the most- and the least-efficient cows during the peripartal period is shown in Fig. 4. Compared with the least-efficient cows, the results indicate that the most-efficient cows had lower overall activities of cellulase (*P* = 0.04), amylase (*P* = 0.02), and protease (*P* < 0.01). In addition, the most-efficient cows had lower (RFI × day, *P* = 0.04) xylanase activity at d 30 postpartum. No day or RFI × day effect (*P* > 0.10) was observed for amylase, cellulase, and protease activity. An RFI × day interaction for xylanase revealed that the most-efficient compared with least-efficient cows had lower (*P* = 0.04) activity at d 30. The interaction effect was also due to lower activity at d 30 relative to other time points.

## Discussion

Although RFI calculations account for BW changes to determine individual feed efficiency independent of changes in BW during the feeding period, published RFI studies in dairy cows have been conducted during mid-lactation where minimal changes in BW occur [31]. Therefore, exploring the physiological differences between the most- and the least-efficient cows during the peripartal period and early lactation when changes in the physiology and metabolism of the dairy cow affect DMI, BW, and FCM appears warranted in the context of assessing the usefulness of RFI-based selection and performance during negative energy and protein balance.

The lack of overall effect of RFI on DMI between wk − 4 to 3 relative to calving or on FCM during the first 60 DIM suggests that RFI per se was not associated with measures of performance during a period when energy and protein balance are at a nadir. It could be possible that the metabolic and immune challenges cows experience during the peripartal period [9, 32] exert some level of control on DMI in the least-efficient cows while the most-efficient cows are able to maintain or decrease DMI within a narrow margin such that marked differences among groups are difficult to detect statistically. The fact that the most-efficient compared with least-efficient cows consumed on average 2.6 kg DMI/d less by wk 4 postpartum indicates that once “stressors” (e.g. proinflammatory cytokines, plasma free fatty acids, hydroxybutyrate) were not impinging on the cow’s ability to achieve their efficiency potential, the most-efficient cows restored the ability to utilize feed more efficiently. *Succinivibrio dextrinosolvens* and *Megaspheara elsdenii* bacteria are involved in propionate production in the rumen [33, 34]. It is well-known that propionate is vital for cow health and milk production since it serves as the main precursor for hepatic gluconeogenesis [35]. Therefore, although ruminal VFA concentration was not measured in this study, we speculate that the greater abundance of *Succinivibrio dextrinosolvens* and *Megaspheara elsdenii* in the most-efficient cows would have increased ruminal propionate production and its availability to the animal for productive purposes. This idea is supported by previous data with mid-lactation dairy cows [36] in which the most-efficient versus least-efficient cows had greater concentrations of propionate and higher propionate:acetate ratio in ruminal fluid. *Megaspheara elsdenii* is also known for its ability to remove lactate from the rumen environment, thus, *Megaspheara elsdenii* plays a vital role in preventing lactic acidosis [37]. In contrast, *Streptococcus bovis* is a lactate producer, and can stimulate lactic acidosis with potential negative effects on the ruminal epithelium [38]. The tendency for the most-efficient cows to have greater relative abundance of *Megaspheara elsdenii* and lower *Streptococcus bovis* from d − 10 to d 60 relative to parturition suggests that the most-efficient cows would have been at a lower risk of developing acidosis.

*Fibrobacter succinogenes* is equipped with various polysaccharide-degrading enzymes able to ferment cellulose primarily to succinic acid and to a lesser extent to acetic and formic acids, rendering this microorganism among the most-active and predominant bacteria involved in fiber degradation in the rumen [39, 40]. Thus, the tendency for greater abundance of this species in the most-efficient cows suggests that fiber digestion and feed utilization contribute to higher feed efficiency. This notion is further supported by data from recent studies [41, 42] indicating that the most-efficient bulls and heifers had higher NDF and DM digestibility compared with the least-efficient cattle.

*Butyrivibrio proteoclasticus* is a fibrolytic bacterium able to breakdown xylan, and produce butyrate [43]. *Prevotella bryantii* is a succinate producer that ferments hemicellulose, pectin, peptides, and amino acids [44–46], whereas *Succinimonas amylolytica* can ferment α-linked glucose molecules such as maltose, dextrin, and starch [47]. Thus, the lower abundance of *Butyrivibrio proteoclasticus*, *Prevotella bryantii*, and *Succinimonas amylolytica* coupled with greater abundance of propionate producers such as *Succinivibrio dextrinosolvens* and *Megaspheara elsdenii* in the most-efficient cows around parturition seems to support the notion that these cows were able to shift the ruminal fermentation pathways in a way that enhanced the production of glucogenic precursors and reduced dietary energy losses. The overall greater relative abundance of *Eubacterium ruminantium*, *Megaspheara elsdenii*, *Prevotella bryantii*, *Selenomonas ruminantium*, and *Streptococcus bovis* after parturition in both RFI groups could be explained in part by the gradual increase in DMI and the switch from a higher-forage diet prepartum to a higher-concentrate diet postpartum [13, 34].

The fact that there was a greater total copy number of the 16S rRNA gene, an indicator of total bacterial density in the rumen, in the most-efficient cows at d − 10 compared with postpartum times indicated that bacterial species other than the 10 evaluated likely proliferated in the most-efficient cows before calving. It is possible that such changes contributed to the improved feed efficiency, e.g., enhancing ruminal fermentation and fiber digestibility. This speculation is in line with recent findings reported by Bonilha et al. [41] who detected greater NDF and DM digestibility in the most-efficient bulls. It is also possible that the greater population of total ruminal bacteria observed in the most-efficient cows would have increased the production and outflow of total microbial mass from the rumen to the small intestine, allowing greater availability of amino acids for absorption and utilization, despite the lower DMI. This idea is supported by previous work demonstrating that stimulating bacterial growth in the rumen increases microbial protein synthesis [48, 49].

The lower activity of amylase, cellulase, and protease in the most-efficient cows from d − 10 to d 60 around parturition could be taken as indication that digestive function in the rumen might have been curtailed. However, several studies reported that efficient beef bulls and heifers had greater digestibility of dry matter, organic matter, NDF, protein, and total digestible nutrients [41, 42]. This apparent discrepancy seems to suggest that microbial digestive enzymes per se may not reflect the actual capacity for feed digestion in feed-efficient cattle. However, the shifts in ruminal bacteria and digestive enzymes observed in the most-efficient cows in the current study could be associated with the reduction in feed intake because decreasing DMI would slow down the rumen passage rate [50], allowing more time for microbes to degrade dietary fiber in the rumen which may explain the improvement in feed digestibility in feed-efficient cattle [41, 42]. Some studies reported that decreasing rumen passage rate is associated with increased energy costs of maintenance for rumen microbes [51, 52], hence, potentially decreasing the production of digestive enzymes.

## Conclusions

Results indicate that better feed efficiency in dairy cows after calving could be attributed, at least in part, to shifts in ruminal bacteria and digestive enzyme activities during the peripartal period and early lactation. Future studies on the association between ruminal parameters such as feed retention time, passage rate, and microbial metabolic functions in cows divergent for RFI during the peripartal period are warranted.

## Additional file

Additional file 1: Preparation of rumen contents for enzyme activities. (DOCX 16 kb)

## Abbreviations

ADF: Acid detergent fiber
BW: Body weight
DMI: Dry matter intake
FCM: Fat-corrected milk
NDF: Neutral detergent fiber
NFC: Non-fiber carbohydrates
RBB + C: Repeated bead-beating plus column
