# Supplementary material for: Association of residual feed intake with abundance of ruminal bacteria and biopolymer hydrolyzing enzyme activities during the peripartal period and early lactation in Holstein dairy cows
Source: J Anim Sci Biotechnol. 2018 May 14;9:43. doi: 10.1186/s40104-018-0258-9 (PMC5956847; doi:10.1186/s40104-018-0258-9)
Supplement: Supplementary file 1 — Preparation of rumen contents for enzyme activities. (DOCX 16 kb) [file 40104_2018_258_MOESM1_ESM.docx]

**Rumen contents preparation for enzyme activity assays**

Frozen rumen contents were thawed at 4^°^C for 24 h then centrifuged at 10,000 *× g* for 1 h at 4^°^C. The supernatant (10 mL) was filtered through an Amicon Ultra-15 10K centrifugal filter by centrifuging at 3,500 *× g* for 2 h at 4^°^C until reaching 1 mL of concentrated sample. The concentrated sample was transferred into a 1.5 mL centrifuge tube then centrifuged at 25,000 *× g* for 20 min at 4^°^C. The clear supernatant was then transferred into a 1.5 mL centrifuge tube and the sample kept at 4^°^C for rumen enzyme activity determination.

**Cellulase and xylanase assay**

The clear supernatant (10 μL) was pipetted into PCR plates containing purified substrate. To determine cellulase activity, 90 μL 1% (wt/vol) Carboxymethylcellulose (CMC) (carboxymethylcellulose sodium salt medium viscosity, Sigma C-4888) in 0.05 M sodium phosphate and 0.15 M sodium chloride buffer (pH 6.0) were added to each well. To determine xylanase activity, 90 μL 1% (wt/vol) wheat arabinoxylan (WAX) (wheat flour medium viscosity, Megazyme P-WAXYM) in 0.05 M sodium phosphate and 0.15 M sodium chloride buffer (pH 6.0) were added to each well. Plates were then incubated at 37^°^C for 3 or 2 h for cellulase and xylanase activities, respectively, to allow enzymatic breakdown of CMC or WAX substrates into their reduced sugars, i.e. glucose or xylose. After incubation, plates were boiled at 100^°^C for 10 min to stop the reaction and cooled to 4^°^C. The reducing sugars were converted into colored product using the para-hydroxybenzoic acid hydrazide (PHBAH) (Sigma H-9882) method as described previously by [Lever (1972)](#_ENREF_14). Briefly, 33.3 μL sample mixture with CMC or WAX was pipetted into PCR plates that contained 100 μL 0.1% (wt/vol) PHBAH in 0.4 M sodium hydroxide and 0.1 M sodium citrate dehydrate. Plates were then boiled at 100^°^C for 10 min. D-(+)-Glucose (Sigma G-7021) and D-(+)-Xylose (Sigma X-1500) standards in 0.05 M sodium phosphate and 0.15 M sodium chloride buffer (pH 6.0) at appropriate concentrations (40-0.31 mM) were used to determine the concentration of liberated glucose or xylose. A blank was kept for each reaction. All assays were performed in triplicate. The absorbance of the emitted color was measured using a spectrophotometer at 410 nm to determine enzyme activities. The units of cellulase and xylanase activities were expressed as μm liberated glucose or xylose per h per mL.

**Amylase assay**

The clear supernatant (6 μL) was pipetted into PCR plates containing

54 µL 1% (wt/vol) starch (starch soluble, Sigma S-9765) and 60 µL 0.1 M sodium phosphate buffer (pH 6.8). Plates were then incubated at 39^°^C for 30 min to allow enzymatic breakdown of starch substrates into glucose. After incubation, plates were boiled at 100^°^C for 7 min to stop the reaction and cooled to 4^°^C. The reducing sugars were converted into colored product using the PHBAH (Sigma H-9882) method as described previously by [Lever (1972)](#_ENREF_14). Briefly, a 33.3 μL sample mixture with starch was pipetted into PCR plates that contained 100 μL 0.1% (wt/vol) PHBAH in 0.4 M sodium hydroxide and 0.1 M sodium citrate dehydrate. Plates were then boiled at 100^°^C for 10 min. D-(+)-Glucose (Sigma G-7021) standards in distilled water of appropriate concentrations (40-0.31 mM) were used to determine the concentration of liberated glucose. The appropriate blank was kept for each reaction. All assays were performed in triplicate. The absorbance of the emitted color was measured using a spectrophotometer at 410 nm to determine amylase activity. The units of amylase activity were expressed as μm liberated glucose per h per mL.

**Protease assay**

The clear supernatant (60 μL) was pipetted into PCR plates containing 60 µL 2% (wt/vol) azocasein (azocasein protease substrate, Sigma A-2765) in 0.1 M citrate phosphate buffer (pH 6.8). Plates were then incubated at 39^°^C for 1 h to allow enzymatic breakdown of azocasein substrate. After incubation, 100 μL of the mixture was incubated with 50 µL 15% (wt/vol) trichloroacetic acid (trichloroacetic acid ACS reagent, ≥ 99.0%., Sigma T-6399) for 30 min on ice to stop the reaction. Azocasein standards in 0.1 M citrate phosphate buffer (pH 6.8) of appropriate concentrations (0.5-0.0039 % wt/vol) were used to quantify the hydrolyzed azocasein. The appropriate blank was kept for each reaction. All assays were performed in triplicate. The absorbance of the developed color was measured using a spectrophotometer at 420 nm to determine enzyme activities. The units of protease activities were expressed as mg hydrolyzed azocasein per h per mL.
